# Effects of Training Programs on Decision-Making in Youth Team Sports Players: A Systematic Review and Meta-Analysis

**DOI:** 10.3389/fpsyg.2021.663867

**Published:** 2021-05-31

**Authors:** Ana Filipa Silva, Rodrigo Ramirez-Campillo, Hugo Sarmento, José Afonso, Filipe Manuel Clemente

**Affiliations:** ^1^N2i, Polytechnic Institute of Maia, Maia, Portugal; ^2^The Research Centre in Sports Sciences, Health Sciences and Human Development (CIDESD), Vila Real, Portugal; ^3^Human Performance Laboratory, Quality of Life and Wellness Research Group, Department of Physical Activity Sciences, Universidad de Los Lagos, Osorno, Chile; ^4^Centro de Investigación en Fisiología del Ejercicio, Facultad de Ciencias, Universidad Mayor, Santiago, Chile; ^5^University of Coimbra, Research Unit for Sport and Physical Activity, Faculty of Sport Sciences and Physical Education, Coimbra, Portugal; ^6^Centre for Research, Education, Innovation and Intervention in Sport, Faculty of Sport, University of Porto, Porto, Portugal; ^7^Escola Superior Desporto e Lazer, Instituto Politécnico de Viana do Castelo, Rua Escola Industrial e Comercial de Nun’Álvares, Viana do Castelo, Portugal; ^8^Instituto de Telecomunicações, Delegação da Covilhã, Lisbon, Portugal

**Keywords:** decision-making, youth sports, psychomotor performance, motor learning, motor skill

## Abstract

**Background:**

The use of dedicated training programs for improving decision-making (DM) in team sports players has grown in the last several years. Approaches such as imagery training, video-based training, or game-based drills are some of the interventions used in youth players in order to improve DM. However, no systematic reviews or meta-analyses have been conducted to summarize the main evidence regarding the effects of these programs on the players and identify the magnitude of the effects compared to control groups.

**Objective:**

This systematic review (with meta-analysis) was conducted to assess the effects of training programs on the DM of youth team sports players.

**Data Sources:**

The data sources utilized were PubMed, PsycINFO, Scopus, SPORTDiscus, and Web of Science.

**Study eligibility criteria:**

The criteria included the following: (i) youth (≤ 18 years old) team sports players with no restriction on sex or competitive level; (ii) players subjected to training programs to develop DM; (iii) control groups; (iv) pre–post outcomes related to tactical behavior, technical execution, reaction, and decision time; and (v) controlled trials.

**Results:**

The database search initially identified 2497 titles. From these, six articles were eligible for the systematic review and meta-analysis. The results showed a significant beneficial effect of DM interventions on tactical behavior (ES = 1.12; *p* = 0.035; *I*^2^ = 80.0%; Egger’s test *p* = 0.066), whereas no significant effect of DM interventions on technical execution was found (ES = 0.74; *p* = 0.180; *I*^2^ = 69.1%; Egger’s test *p* = 0.873).

**Conclusion:**

The DM interventions were significantly effective in improving tactical behavior in youth team sports players independently from the number of sessions to which players were exposed. In addition, DM interventions were significantly effective in improving technical execution. However, the results should be carefully interpreted due to the heterogeneity of the articles’ overall methodological quality. Future DM interventions should consider using combined approaches that allow players to develop both tactical behavior and technical execution.

## Introduction

Team sports can be characterized as a dynamic system in which decisions made by players are crucial to improvements in individual and collective performance (McGarry et al., 2002). During games, players are continuously challenged to perceive the environment and adjust their behaviors in accordance with their teammates’ behaviors and contextual factors (Araujo and Davids, 2016). Therefore, various decisions are made by players resulting from the perception–action cycle and influenced by functional constraints (Araújo et al., 2019). That means that there is there is no single or better decision, as the game is a dynamic and open system (Araújo et al., 2006; Davids and Araújo, 2010).

Decision-making (DM) is the ability to choose the most functional and effective option(s) from a vast array of possibilities emerging from different game scenarios (Hastie, 2001). From the point of view of ecological dynamics, two main levels of analysis can be considered (Araújo et al., 2006): (i) agent–environment interactions (in which the player acts to detect information) and (ii) temporal evolution of the player’s behavior (in which changes in few key variables promote variations in the player’s behaviors). Therefore, DM is a complex and dynamic process that depends on the interrelationships between the player(s) and the environment.

In sports, the level of expertise of the players seems to play an important role in the quality of DM during games or visualization of game scenarios (Scharfen and Memmert, 2019). In fact, it was found that experts made their decisions faster, better, and more intuitive than less experts (Raab and Laborde, 2011). In a recent systematic review (Silva et al., 2020), it was suggested that expert and older players make more accurate decisions, present more developed tactical knowledge, and engage in more effective tactical behaviors than novice players. The level of expertise does not result exclusively from age or years of practice but also from the quality of practice (Williams and Ford, 2008). In addition, it was found that in low-complexity game situations, implicit learners, i.e., automatic acquisition of knowledge, are superior, but in high-complexity situations, explicit learners (intentional acquisition that results in verbalized knowledge) were better (Raab, 2003). The athlete’s creativity also seems to play an important role; as in the Roca et al. (2018) study, the most-creative players produced more appropriate, original, flexible, and fluid decisions compared to the least-creative players. For this reason, task representativeness should be part of training scenarios that aim to increase the capacity of players to act in dynamic scenarios that help them develop an athlete–environment relationship (Travassos et al., 2013).

Making expert players is part of long-term development programs that start in youth categories. Considering the importance of implementation strategies for youth players in increasing the quality of their decisions, and due to the importance of DM in sports performance, experimental approaches have tested interventions that may help to develop the capacity to perceive the environment and make appropriate decisions (Panchuk et al., 2018; Praxedes et al., 2018; Gil-Arias et al., 2019). Nevertheless, it should be noted that the main part of the human brain that is involved in executive functions as DM, the left dorsolateral prefrontal cortex, continues to develop throughout adolescence into early adulthood (Fuster, 2002; Mukherjee et al., 2002). Therefore, it is important to determine how DM-based programs can be implemented for developing youth players to make them better at making decisions and understanding the reality of the dynamics of the game.

Numerous studies have analyzed DM using different methodologies. They identified seven different tools to train DM (Vickers, 2003), which include variable practice, random practice, bandwidth feedback, questioning, video feedback, hard-first instruction and modeling, and external focus instruction. From those, video training has been highlighted as an important tool to develop exposure to relevant perceptual cues and knowledge about opposition athletes and their tactics (Araújo et al., 2005). Following this, it was reported by athletes that video training, organized training, and watching games on television were the best strategies to develop perceptual and DM skills (Baker et al., 2003). Indeed, various aspects involved in DM could be analyzed; therefore, they should be selected regarding the demands of the sport to be analyzed (Cotterill and Discombe, 2016). Nevertheless, the task needs to be kept as ecologically valid as possible. Following Cotterill (2014), three specific points should be considered when conducting interventions/trainings: (i) conscious cognitive (developing an understanding of past experience, tactical awareness, and the individual players’ predispositions and tendencies), (ii) perception–action coupling, and (iii) abort and reset (rapidly respond to changes in the environment).

There is growing evidence of the benefits of training interventions for developing DM in youth athletes. Recent systematic reviews have summarized different pieces of evidence regarding DM in youth sports (Travassos et al., 2013; Scharfen and Memmert, 2019; Silva et al., 2020), mainly focusing on comparisons between expert and novice players. Although several reviews are available, none of them have tested the effectiveness of DM programs against control groups. Additionally, previous studies testing the effectiveness of DM programs have typically used small samples; thus, a meta-analysis is needed to pool the data. Finally, one question remains: How effective are DM training interventions compared to control groups? The answer to this question may help identify the potential value of DM interventions for the improvement of DM in youth athletes. Therefore, the aim of this systematic review and meta-analysis (SRMA) is intended to assess the effects of training interventions on the DM (tactical behavior and technical execution) of youth team sports players.

## Methods

The present SRMA followed the Cochrane Collaboration guidelines (Green and Higgins, 2005). The systematic review strategy was conducted according to PRISMA (Preferred Reporting Items for Systematic Reviews and Meta-analyses) guidelines (Moher et al., 2009). The PICOS approach (Population, Intervention, Comparator, Outcomes, Study design) (Moher et al., 2015) was followed to define the inclusion criteria (Table 1). The protocol was registered with the International Platform of Registered Systematic Review and Meta-Analysis Protocols with the number INPLASY2020100082 and the DOI number 10.37766/inplasy2020.10.0082.

**TABLE 1:** PICOS approach.

PICOS components	Details
Population	Youth (10–18 years old) team sports players, not limited to expertise level
Intervention	Players subjected to training programs for developing decision-making
Comparator	Control groups
Outcomes	Tactical behavior, technical execution, reaction, and decision time
Study design	Controlled trials

### Information Sources

A comprehensive computerized search of the following electronic databases was performed: (i) PubMed, (ii) PsycINFO, (iii) Scopus, (iv) SPORTDiscus, and (v) Web of Science. The searching process for relevant publications had no restriction regarding year of publication and included articles retrieved until October 21, 2020. Keywords and synonyms were entered in various combinations in title, abstracts, and keywords: (youth OR young) AND (“decision making” OR decision^∗^ OR “decision training”) AND (“team sport” OR football OR soccer OR futsal OR handball OR volleyball OR basketball OR hockey OR rugby OR cricket OR “water polo” OR lacrosse OR softball OR korfball OR “American football”).

### Eligibility Criteria

Inclusion criteria for this systematic review and meta-analysis were as follows: (i) youth (between 10—possible onset of puberty—and 18 years old—until adulthood) team sports players with no restriction of sex, expertise level, or competitive level; (ii) players subjected to training programs for developing DM (e.g., imagery, video-based, and drill-based games) with no restrictions for total program duration; (iii) control groups; (iv) pre–post outcomes related to tactical behavior, technical execution, reaction, and decision time; (v) controlled trials; and (vi) original peer-reviewed articles written in English. Studies were excluded on the basis that they (i) were observational analytic designs; (ii) included other sports than team sports; and (iii) were review articles, letters to the editor, errata, invited commentaries, or conference abstracts. Exclusion criteria were as follows: (i) adults (> 18 years old) and/or players from sports other than team sports; (ii) interventions not related to DM; (iii) no control group or active controls (additional interventions to regular training sessions); (iv) outcomes not related to DM, or no pre–post data reported; (v) non-controlled trials; and (vi) non-original peer-reviewed articles or articles written in language other than English.

### Extraction of Data

A data extraction sheet conceived in Microsoft Excel (Microsoft Corporation, Redmon, WA, United States) was made based on Cochrane Consumers and Communication Review Group’s data extraction template (Collaboration, 2016). The sheet was used to assess inclusion requirements and subsequently tested on 10 randomly selected studies (i.e., pilot testing). Two of the authors (FMC and JA) conducted the process. Any disagreement regarding study eligibility was resolved in a discussion between the authors. Full text articles excluded, with reasons, were recorded. All the records were stored in the sheet.

### Data Items

Regarding the included studies, the outcomes could be grouped in (i) action or reaction time (s), for those studies testing the effects of intervention on time-based DM tests; (ii) overall success in technical execution, for those studies testing the effects on the accuracy of technical actions; and (iii) overall success in tactical behavior, for those studies comparing the intervention effects on the number or percentage of tactical behaviors performed. For all the included studies, the pre-intervention and post-intervention data were collected. Cases as retention data (after post-intervention) were not extracted.

Additionally, the following information was extracted from the included studies: (i) number of participants (*n*), age (years), and sex; (ii) the type of intervention (e.g., imagery, video-based, and drill-based games); (iii) period of intervention (number of weeks) and number of sessions per week (n/w); and (iv) characteristics of intervention (e.g., tasks, process).

### Assessment of Methodological Quality

Version 2 of the Cochrane risk-of-bias tool for randomized trials (RoB2) (Sterne et al., 2019) was used to assess the risk of bias in the included randomized-controlled trials. Five dimensions are inspected in this assessment tool: (i) bias arising from the randomization process; (ii) bias due to deviations from intended interventions; (iii) bias due to missing outcome data; (iv) bias in measurement of the outcome; and (v) bias in selection of the reported result. Using RoB2, a qualitative synthesis was performed. Two of the authors (JA and HS) independently assessed the risk of bias. Any disagreement in the rating was resolved through discussion and by a third author (FMC).

The Cochrane risk of bias in non-randomized studies of interventions (ROBINS-I) was used to assess the risk of bias in included non-randomized intervention studies (Sterne et al., 2016). Three domains are analyzed in this assessment tool: (i) pre-intervention (bias due to confounding; bias in selection of participants into the study); (ii) at intervention (bias in classification of interventions); and (iii) post-intervention (bias due to deviations from intended interventions, bias due to missing data, bias in measurement of outcomes, and bias in selection of the reported results). Two of the authors (JA and HS) independently assessed the risk of bias. Any disagreement in the rating was resolved through discussion and by a third author (FMC).

### Summary Measures, Synthesis of Results, and Publication Bias

Although two studies can be used in meta-analyses (Valentine et al., 2010), considering reduced sample sizes are common in the sports science literature (Abt et al., 2020), analysis and interpretation of results in this systematic review and meta-analysis were only conducted in the case of at least three study groups provided baseline and follow-up data for the same measure. Pre-training and post-training means and standard deviations (SD) for dependent variables were used to calculate effect sizes (ES; Hedges’s *g*) for each outcome in the intervention and control groups. Data were standardized using post-intervention SD values. The random-effects model was used to account for differences between studies that might impact the intervention effect (Deeks et al., 2008; Kontopantelis et al., 2013). The ES values are presented with 95% confidence intervals (CI). Calculated ES were interpreted using the following scale: < 0.2, trivial; 0.2–0.6, small; > 0.6–1.2, moderate; > 1.2–2.0, large; > 2.0–4.0, very large; > 4.0, extremely large (Hopkins et al., 2009). Heterogeneity was assessed using the *I*^2^ statistic, with values of < 25%, 25–75%, and > 75% considered to represent low, moderate, and high levels of heterogeneity, respectively (Higgins and Thompson, 2002). The risk of bias was explored using the extended Egger’s test (Egger et al., 1997). To adjust for publication bias, a sensitivity analysis was conducted using the trim and fillmethod (Duval and Tweedie, 2000), with L0 as the default estimator for the number of missing studies (Shi and Lin, 2019). All analyses were carried out using the Comprehensive Meta-Analysis software (version 2; Biostat, Englewood, NJ, United States). Statistical significance was set at *p* ≤ 0.05.

### Analysis of Moderator Variables

The potential effects of moderator variables were assessed by executing a sub-group analysis. A random-effects model was used for testing the effect of longer (>12 sessions) and shorter (<12 sessions). The threshold value of 12 sessions was defined based on the median of sessions of all the included experimental programs.

## Results

### Study Identification and Selection

**FIGURE 1:**
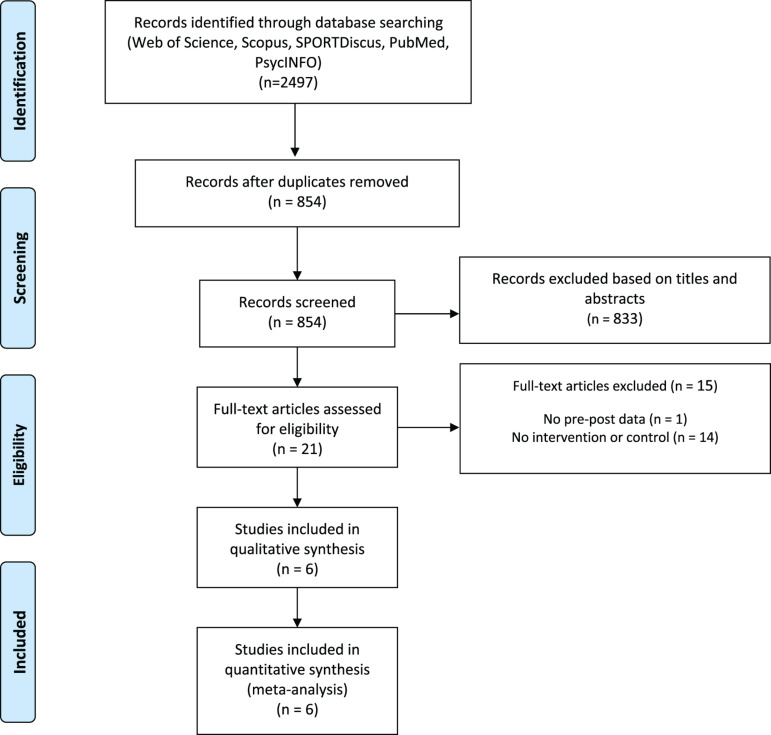
PRISMA flow diagram highlighting the selection process for the studies included in the current systematic review.

The database search identified 2497 titles. These studies were then exported to reference manager software (EndNote X9, Clarivate Analytics, Philadelphia, PA, United States). Duplicates (1643 references) were subsequently removed either automatically or manually. The remaining 854 articles were screened for their relevance based on titles and abstracts, resulting in the removal of a further 833 studies. The full texts of the remaining 21 articles were examined diligently; 15 were excluded due to a number of reasons (Figure 1). The six studies included in the meta-analysis provided mean and standard deviation for pre- and post-intervention data for at least one main outcome.

### Study Characteristics

The characteristics of the six studies included in this systematic review and meta-analysis can be found in Table 2. Additionally, the details of the DM training programs can be found in Table 3. The included controlled studies involved six individual experimental groups and 56 participants, and 52 participants in the six control groups. Four of the included studies were exclusively conducted in boys (Hohmann et al., 2016; Práxedes et al., 2016; Gil-Arias et al., 2019; Fortes et al., 2020) and one was exclusively conducted in girls (Gil-Arias et al., 2016), while one study included boys and girls (Panchuk et al., 2018). Two studies included basketball players (Panchuk et al., 2018; Gil-Arias et al., 2019), two studies included volleyball players (Gil-Arias et al., 2016; Fortes et al., 2020), one study included handball (Hohmann et al., 2016), and another study included soccer players (Práxedes et al., 2016). In the six included studies, a measure of overall success in tactical behavior was extracted. In three of the included studies (Práxedes et al., 2016; Panchuk et al., 2018; Gil-Arias et al., 2019), a measure of overall success in technical execution was extracted. Since just one of the included studies reported data about reaction time (Hohmann et al., 2016), the outcome was not included in the meta-analysis.

**TABLE 2:** Characteristics of the included studies and outcomes extracted.

Study	*N*	Mean age (yo)	Experience (y)	Sex	Team Sport	Training level	Design	Sig. Dif. Baseline	Outcomes extracted	Tests or tools used	Measure used
Fortes et al. (2020)	Intervention (*n* = 17) Control (*n* = 16)	Intervention: 15.6 ± 1.9 Control: 15.6 ± 1.8	ND	M	Volleyball	State level	RCT	No	Overall success in tactical behavior	Game Performance Evaluation Tool	DM index (passing)
Gil-Arias et al. (2016)	Intervention (*n* = 4) Control (*n* = 4)	Intervention: 15.0 ± 0.8 Control: 14.5 ± 0.6	Intervention: 3.8 ± 1.0 Control: 4.3 ± 0.5	W	Volleyball	Regional level	CT	No	Overall success in tactical behavior	Game Performance Assessment Instrument	DM in attack action
Gil-Arias et al. (2019)	Intervention (*n* = 5) Control (*n* = 6)	Intervention: 12.4 ± 0.5 Control: 12.7 ± 0.5	Intervention: 5.2 ± 0.9 Control: 12.7 ± 0.5	M	Basketball	ND	RCT	No	Overall success in tactical behavior Overall success in technical execution	The French and Thomas (1987) observation instrument	General DM General skill execution
Hohmann et al. (2016)^a^	Intervention (*n* = 10) Control^b^ (*n* = 10)	Overall: 14.9 ± 0.8	ND	M	Handball	Regional level	CT	ND	Overall success in tactical behavior	Video sequences and comparison to national you team coaches	Percentage correct for best options
Panchuk et al. (2018)	Intervention (*n* = 11; 5 men and 6 women) Control (*n* = 7; 4 men and 3 women)	Overall: 17.0 ± 0.6	ND	W and M	Basketball	Elite youth	CT	ND	Overall success in tactical behavior Overall success in technical execution	Immersive test score Total small-sided game score	Immersive test score Total small-sided game score
Práxedes et al. (2016)	Intervention (*n* = 9) Control (*n* = 9)	Overall: 10.7 ± 0.6	Intervention: 4.9 ± 0.8 Control: 4.8 ± 0.1	M	Soccer	ND	CT	No	Overall success in tactical behavior Overall success in technical execution	Game Performance Evaluation Tool	DM skills (pass) Execution skills (pass)

*N, number of participants in the study; Yo, years old; Y, years; M, men; W, women; h, hour; CG, control group; Sig. Dif. Baseline, significant differences at baseline; ND, not-described; ^a^, only study 2 of the article was included; ^b^, passive control; DM, decision-making; CT, controlled trial; RCT, randomized controlled trial; ^a^, data from study 2.*

**TABLE 3:** Characteristics of intervention programs in the included studies.

Study	Duration (w)	Sessions/week (*n*)	Total sessions (*n*)	Type of intervention	Time of intervention per session (min)	Characteristics of intervention
Fortes et al. (2020)	8	3	24	Imagery-based intervention	10 min	Before imagery training, players watched succeeding volleyball passes. Players were requested to imagine themselves executing passes during a competitive event. The following order was established: (i) construct imagery situation in the first person; (ii) imagine the task with speed close to reality; (iii) imagine positive situations during a competition; and (iv) generate emotions like in a competition.
Gil-Arias et al. (2016)	11	2	22	Video-based and questioning-based interventions	45 min	For each session, three processes were made: (1) viewing the attack action; (2) self-analysis and player’s reflection; and (3) combined analysis player-expert. A total of 44 attack actions were analyzed by the players in the experimental group. After a 6 vs. 6 format of play, the players left to be submitted to a decision training program.
Gil-Arias et al. (2019)	11	1	11	Video-based and questioning-based interventions	45 min	For each session, three processes were made: (1) viewing the attack action; (2) self-analysis and player’s reflection; and (3) combined analysis player-expert. For each session, a set of 6 actions were analyzed for each player.
Hohmann et al. (2016)^a^	6	1	6	Video-based intervention	30 min	Three-dimensional video analysis was implemented. Sixty-four decision tasks per session were implemented. Typical attacking and defensive scenarios were used in the videos. Players provided their feedbacks about the correct solutions.
Panchuk et al. (2018)	3	Women (*n* = 3.3) Men (*n* = 4)	Women (*n* = 10) Men (*n* = 12)	Video-based intervention	5 min	Immersive video clips (360° video footage), custom designed were implemented in basketball players. The video played until the moment of the decision, in which the player need to select the answer.
Práxedes et al. (2016)	12	2	21	Game-based intervention	60 min	Teaching games for understanding was implemented in the experimental group. Four modified games were used in each session. Small-sided and conditioned games respected the pedagogical principles of representation and exaggeration. Questions were also prepared for each modified game.

*w, number of weeks; n, number; min: minutes; ^a^, data from study 2.*

### Methodological Quality

The two randomized controlled trials (Gil-Arias et al., 2019; Fortes et al., 2020) included in this study were analyzed with RoB, and the risk of bias can be found in Table 4.

**TABLE 4:**
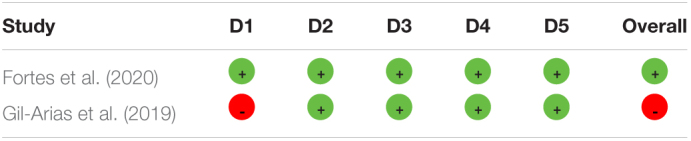
Assessment of RoB.

*D1, randomization process; D2, Deviations from the intended interventions; D3, Missing outcome data; D4, Measurement of the outcome; D5, Selection of the reported result. Green, low risk; Yellow, Some concerns; Red, high risk; NA, not applicable.*

Among the non-randomized studies, intervention studies assessed for the risk of bias (Table 5), three (Hohmann et al., 2016; Práxedes et al., 2016; Panchuk et al., 2018) presented overall critical risk of bias, while one presented serious risk of bias (Gil-Arias et al., 2019).

**TABLE 5:**
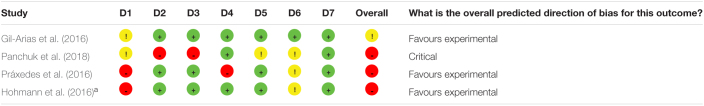
Assessment of ROBINS-I.

*^a^, Only study 2 of this article was included; D1, reaching risk of bias judgements for bias due to confounding; D2, reaching risk of bias judgments in selection of participants into the study; D3, reaching risk of bias judgments for bias in classification of interventions; D4, reaching risk of bias judgments for bias due to deviations from intended interventions; D5, reaching risk of bias judgements for bias due to missing data; D6, reaching risk of bias judgements for bias in measurement of outcomes; D7, reaching risk of bias judgments for bias in selection of the reported result; Green, low risk; Yellow, moderate/serious risk; Red, critical risk.*

### Intervention vs. Control on Tactical Behavior

A summary of the included studies and results of tactical behavior reported before and after training programs are provided in Table 6.

**TABLE 6:** Summary of the included studies and results of tactical behavior before and after intervention.

Study	Group	*N*	Before Mean ± SD	After Mean ± SD	After - before (%)
Gil-Arias et al. (2016)	Intervention	4	47.2 ± 0.1	67.2 ± 0.1	42.4
Gil-Arias et al. (2019)	Intervention	5	72.8 ± 6.6	87.9 ± 1.8	20.7
Panchuk et al. (2018)^a^	Intervention	5	46.2 ± 5.4	50.2 ± 4.5	8.7
Panchuk et al. (2018)^b^	Intervention	6	38.3 ± 3.1	45.7 ± 4.9	19.3
Práxedes et al. (2016)	Intervention	9	0.75 ± 0.15	0.85 ± 0.14	13.3
Hohmann et al. (2016)^c^	Intervention	10	60.0 ± 3.0	66.0 ± 4.0	10.0
Fortes et al. (2020)	Intervention	17	0.67 ± 0.07	0.75 ± 0.08	11.9
Gil-Arias et al. (2016)	Control	4	34.8 ± 0.2	46.4 ± 0.1	33.3
Gil-Arias et al. (2019)	Control	6	76.9 ± 8.3	73.3 ± 5.7	−4.7
Panchuk et al. (2018)^a^	Control	4	50.5 ± 5.8	50.8 ± 2.2	0.6
Panchuk et al. (2018)^b^	Control	3	36.0 ± 4.4	45.7 ± 2.5	26.9
Práxedes et al. (2016)	Control	9	0.76 ± 0.15	0.64 ± 0.23	−15.8
Hohmann et al. (2016)^c^	Control	10	57.0 ± 3.0	61.0 ± 2.0	7.0
Fortes et al. (2020)	Control	16	0.66 ± 0.08	0.65 ± 0.08	−1.5

*^a^, men; ^b^, women; ^c^, only study 2 of this article was included.*

**FIGURE 2:**
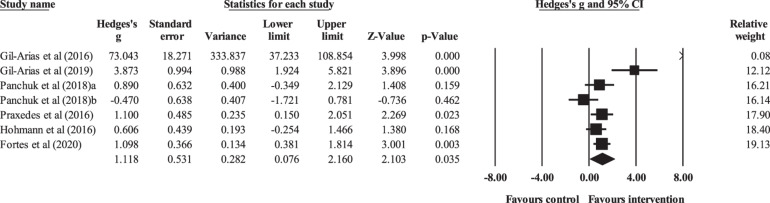
Forest plot of changes in tactical behavior, in youth athletes from team sports participating in decision-making training (intervention) compared to controls. Values shown are effect sizes (Hedges’s *g*) with 95% confidence intervals (CI). The size of the plotted squares reflects the statistical (relative) weight of the study.

Six controlled studies provided data for tactical behavior, involving seven experimental and six control groups (pooled *n* = 108). There was a significant effect of DM interventions on tactical behavior (ES = 1.12; 95% CI = 0.08 to 2.16; *p* = 0.035; *I*^2^ = 80.0%; Egger’s test *p* = 0.066; Figure 2). The relative weight of each study in the analysis ranged from 0.1% to 19.1%.

**FIGURE 3:**
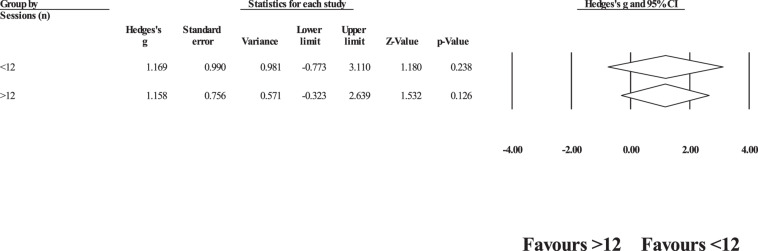
Sub-group analyses testing the effects of programs that were longer and shorter than 12 sessions.

No significant sub-group difference in tactical behavior (*p* = 0.993) was found when DM training interventions < 12 sessions (three experimental groups; ES = 1.17; 95% CI = −0.77 to 3.11; within-group *I*^2^ = 85.3%) were compared to DM training interventions > 12 sessions (four experimental groups; ES = 1.16; 95% CI = −0.32 to 2.64; within-group *I*^2^ = 80.8%; Figure 3).

**FIGURE 4:**
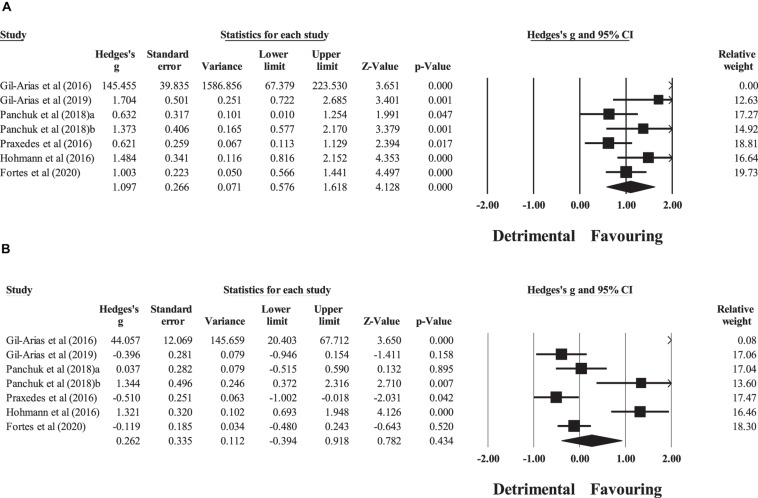
Forest plot of within-group pre–post intervention changes in tactical behavior, in youth athletes from team sports participating in **(A)** decision-making training and **(B)** control condition. Values shown are effect sizes (Hedges’s *g*) with 95% confidence intervals (CI). The size of the plotted squares reflects the statistical (relative) weight of the study.

Six studies provided data for within-group pre–post meta-analyses of tactical behavior for the experimental groups (pooled *n* = 56). There was a significant favoring effect of DM training on tactical behavior (ES = 1.10; 95% CI = 0.58 to 1.62; *p <* 0.001; *I*^2^ = 72.1%; Egger’s test *p* = 0.010; Figure 4A). The relative weight of each study in the analysis ranged from 0.0% to 19.7%. The sensitivity analysis indicated an adjusted value of ES = 0.88, 95% CI = 0.32 to 1.44.

Six studies provided data for within-group pre–post meta-analyses of tactical behavior for the control groups (pooled *n* = 52). There was a non-significant effect of the control condition on tactical behavior (ES = 0.26; 95% CI = −0.39 to 0.92; *p* = 0.434; *I*^2^ = 86.4%; Egger’s test *p* = 0.052; Figure 4B). The relative weight of each study in the analysis ranged from 0.1% to 18.3%.

### Intervention vs. Control on Technical Execution

A summary of the included studies and results of technical execution reported before and after training programs are provided in Table 7.

**TABLE 7:** Summary of the included studies and results of technical execution before and after intervention.

Study	Group	*N*	Before Mean ± SD	After Mean ± SD	After - before (△%)
Gil-Arias et al. Gil-Arias et al. (2019)	Intervention	5	64.2 ± 8.1	75.1 ± 3.3	17.0
Panchuk et al. Panchuk et al. (2018)^a^	Intervention	5	20.2 ± 9.3	26.1 ± 6.3	29.2
Panchuk et al. Panchuk et al. (2018)^b^	Intervention	6	22.5 ± 8.1	23.0 ± 8.1	2.2
Praxedes et al. Práxedes et al. (2016)	Intervention	9	0.62 ± 0.18	0.72 ± 0.13	16.1
Gil-Arias et al. Gil-Arias et al. (2019)	Control	6	65.2 ± 9.5	67.4 ± 3.6	3.4
Panchuk et al. Panchuk et al. (2018)^a^	Control	4	15.3 ± 10.6	16.5 ± 6.3	7.8
Panchuk et al. Panchuk et al. (2018)^b^	Control	3	23.8 ± 5.3	30.5 ± 4.9	28.2
Praxedes et al. Práxedes et al. (2016)	Control	9	0.64 ± 0.16	0.55 ± 0.27	−14.1

*^a^, men; ^b^, women.*

**FIGURE 5:**
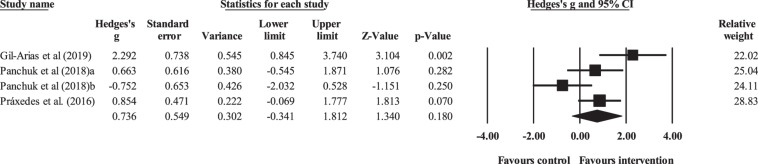
Forest plot of changes in technical execution, in youth athletes from team sports participating in decision-making training (intervention) compared to controls. Values shown are effect sizes (Hedges’s *g*) with 95% confidence intervals (CI). The size of the plotted squares reflects the statistical (relative) weight of the study.

Three controlled studies provided data for technical behavior, involving four experimental and three control groups (pooled *n* = 47). There was a non-significant effect of DM interventions on technical execution (ES = 0.74; 95% CI = −0.34 to 1.81; *p* = 0.180; *I*^2^ = 69.1%; Egger’s test *p* = 0.873; Figure 5). The relative weight of each study in the analysis ranged from 22.0% to 28.8%.

**FIGURE 6:**
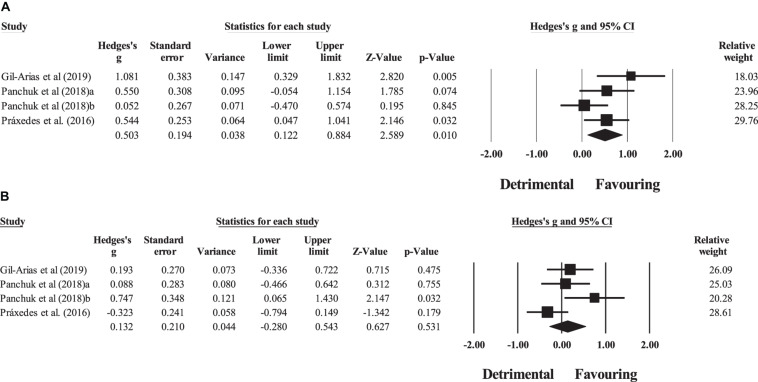
Forest plot of within-group pre–post intervention changes in technical behavior, in youth athletes from team sports participating in **(A)** decision-making training and **(B)** control condition. Values shown are effect sizes (Hedges’s *g*) with 95% confidence intervals (CI). The size of the plotted squares reflects the statistical (relative) weight of the study.

Three studies provided data for within-group pre–post meta-analyses of technical behavior (pooled *n* = 25). There was a significant favoring effect of DM training on technical behavior (ES = 0.50; 95% CI = 0.12 to 0.88; *p* = 0.010; *I*^2^ = 41.7%; Egger’s test *p* = 0.270; Figure 6A). The relative weight of each study in the analysis ranged from 18.0% to 29.8%.

Three studies provided data for within-group pre–post meta-analyses of technical behavior (pooled *n* = 22). There was a non-significant effect of the control condition on technical behavior (ES = 0.13; 95% CI = −0.28 to 0.54; *p* = 0.531; *I*^2^ = 54.9%; Egger’s test *p* = 0.039; Figure 6B). The relative weight of each study in the analysis ranged from 20.3% to 28.6%. The sensitivity analysis indicated an adjusted value of ES = −0.03, 95% CI = −0.48 to 0.43.

## Discussion

This systematic review (with meta-analysis) aimed to analyze the effects of DM-based programs on tactical behavior and the technical execution of youth team sports players. Meta-analytical comparison to control groups was performed. Briefly, tactical behavior was significantly improved by DM programs, whereas technical execution did not provide any meaningful benefits compared to control groups.

### Intervention Versus Control on Tactical Behavior: Summary of Evidence

Tactical skills are linked to the ability of a player to make and execute an appropriate decision in any given situation according to game constraints (Gréhaigne et al., 1995). The notions of perception, anticipation, and decision are key elements of tactical thinking (Gréhaigne et al., 2001). Regarding the included interventions, tactical behavior showed significant positive results. These improvements could be because the interventions led the athletes to focus more on the existing conditions (Gil-Arias et al., 2016), which facilitated perception. In fact, concerning the included studies, the methodology that had the greatest impact on DM were the studies that applied both video-feedback and questioning (Gil-Arias et al., 2016, 2019). Those types of intervention programs are effective mainly because athletes keep their attention focused on more relevant stimuli, recover the best response from their long-term memory, and choose the most effective response according to the objectives of the game (Vickers, 2007). Nevertheless, those scenarios should vary enough (e.g., quantity and type of decision) to maintain the player engagement. Thus, stimuli should be specific to the group that they are designed (e.g., female athletes should view footage of female athletes within the training footage) (Panchuk et al., 2018). Finally, 3D video training was found to be more effective than 2D video, but only in reference to decision time and not decision quality (Hohmann et al., 2016). Those results suggest that cognitive tools should be included in training to improve sports expertise in young ages.

Adolescence is a crucial stage of development—the transition from childhood to adulthood is marked by rapid changes in physical, cognitive, social, and affective development (Mann et al., 1989). To achieve a high level of expertise in sports, mental representations and the cognitive processes that occur between the interpretation of this stimulus and the response selection are crucial (Belling et al., 2015). However, it is during adolescence that DM-dependent abilities, such as perception, attention, anticipation, and working memory, are developed (Memmert, 2010; Albert and Steinberg, 2011; Araújo et al., 2015). Moreover, it has been identified that by around 15 years of age, adolescents have achieved a reasonable level of competence of major factors of DM components (Albert and Steinberg, 2011). This could be the explanation why, in the Panchuk et al. (2018), the results with boys around 17 years presented the lowest improvements in DM. Nevertheless, in opposition, girls with the same age presented an improvement of about 19.3%, the third largest of all included studies. It could be expected that 12 sessions of DM training would not be enough to develop and observe improvements in that capability. However, the results showed that no differences were observed between interventions with less than 12 sessions and those with more than 12 sessions. Nevertheless, regular DM training should be considered—in the study by Gil-Arias et al. (2016), 4 weeks after the end of the intervention, knowledge retention was not observed. Although it was not included, information about athletes’ experience in all the included studies, following the ecological approach, various exploratory actions of perceptual systems are required for perception to occur; i.e., due to the training and experience, the perceptual learning promotes the process of becoming attuned (better able to differentiate information) (Araújo et al., 2019). Those results together could indicate that more important than age could be the methodology used and whether tasks match what athletes really need to improve in their DM.

Regarding the interventions analyzed, it was possible to register the effectiveness of the training as no effects on the control groups were observed between the pre- and post-test. Conversely, a favorable effect of DM training was observed in the training groups. Indeed, questioning or performing imaginary training sessions led athletes to focus their attention on relevant aspects of the game situation (e.g., location of teammates, the positions of players on the opposite team) to carry out a more suitable interpretation of the tendencies, strengths, and weaknesses of the opponent (Mann et al., 2007; Murphy et al., 2018). As a result, athletes learned to select the most appropriate options to make it difficult for the opponents to play their own game (Gil-Arias et al., 2019). Therefore, given the relevance of DM in sports, it has been suggested that there is a need to develop training programs aimed at improving players’ DM and to design representative tasks that include essential aspects of the context of the game (Marasso et al., 2014).

In five of the six studies included in the present study, some kind of video was used for DM training. The study by Fortes et al. (2020) was the only one in which an imagery strategy was applied. This technique is based on the creation of mental images from sensory processes stored in memory, which can be accessed without external stimuli (Battaglia et al., 2014). This technique was also considered a valuable method to improve DM, and it has been shown that neural circuits activated when we perform an action are the same ones that are activated when we imagine it (Roberta et al., 2020). Nonetheless, given that DM relies on the brain’s ability to extract contextual information from the visual scene (Gil-Arias et al., 2016; Romeas et al., 2016), the use of a video that simulates a real practice seems fundamental for DM training. In fact, by using video feedback, athletes can observe and identify the opposite team’s strengths and weaknesses while also improving their recognition of contextual factors (Groom et al., 2011; Nelson et al., 2014). However, to be effective, that observation should be accompanied by an expert to guide athletes’ attention to the most relevant features (Vickers, 2007). In addition, Hohmann et al. (2016) suggested that a 3D video presentation is more effective in improving decision time than a 2D video or a presentation with a tactic board.

Decision-making involves many cognitive processes, such as information searching and processing, problem-solving, judgment, learning, and memory (Mann et al., 1989). In fact, in a review and meta-analysis focused on volleyball, it was found that a training program-based cognitive perspective led to a significant improvement in DM through memory-related processes, compared to those that conduct regular trainings (Suárez et al., 2020). In the field of sports, DM should generate the best option in the shortest time possible. In terms of tactical skills, training by video (especially in 3D and with a representation of the real practice) and training by imagery seem to have a positive effect on improving DM in the adolescent population. Nevertheless, this kind of training has to be guided by an expert or mentor to have a significant positive effect (i.e., the simple visualization of videos/game situations does not guarantee DM improvements).

### Intervention vs. Control on Technical Execution: Summary of Evidence

Few studies have analyzed technical execution when compared to tactical skills evaluations (only three studies, with four experimental and three control groups). The analysis showed no significant effects of the DM interventions. It is known that a better understanding of the game leads to better tactical behaviors and tactical DM during competitions, which enable athletes to achieve a high level of performance (Costa et al., 2010). However, to perform successfully, athletes should present well-developed skills, not only in a declarative (“what to do”) sense but also in a procedural (“doing it”) sense. Therefore, DM training could help athletes clarify sports-specific knowledge to pick up the most important features to pay attention to during a game, but practice is also key in developing skills to apply those intentions.

When intra-group changes were analyzed, a significant effect of DM training sessions was observed in the experimental groups. However, this result was not unanimous among the included studies, with two of them showing positive effects on technical execution (Panchuk et al., 2018; Gil-Arias et al., 2019) and another presenting no improvements (Práxedes et al., 2016). As only three studies analyzed technical execution, these results should be carefully considered. Nevertheless, these differences in the influence on technical execution could be due to the athletes’ level of expertise, since those studies vary among 10 (Práxedes et al., 2016), 12 (Gil-Arias et al., 2019), and 17 (Panchuk et al., 2018) years of age. In fact, it has been indicated that the experience allows the creation of favorable conditions for an adaptation of brain structures to external stimuli, promoting neuroplasticity (Roberta et al., 2020). Moreover, the sample sizes were always small (less than 10 players), which could influence the results. Therefore, more studies in this field should be done to better clarify the possible benefits of learning.

Taken together, these results suggest that DM training might have an indirect effect on the quality of technical execution, as athletes who have a greater capacity to satisfactorily solve tactical situations are also able to modify and adapt their motor execution more effectively. Nevertheless, the study by Panchuk et al. (2018), which included male and female athletes, suggested that sex influenced the testing and training responses, as the male control group did not show the same pattern of change as the female control group. However, in this study, a balance between females and males was not observed, and other methodological issues should be considered as the smallest number of tests and number of training sessions performed by females.

### Limitations, Future Research, and Practical Applications

One limitation of this systematic review and meta-analysis is that it only included articles written in English. Therefore, it is possible that relevant publications written in a language other than English may have been overlooked. Also, most of the studies follow the assumption that DM is based on the internalized knowledge structures (operating as inference engines) to choose the best decision, or the decision that best fits that context. However, following the ecological approach, DM is a dynamic complex process that is based on the context in which it operates. In addition, randomized and non-randomized studies were included even though both types presented no significant differences between groups at baseline. However, this meta-analysis should be carefully interpreted due to the heterogeneity of the articles’ overall methodological quality. Finally, the meta-analysis included any type of DM intervention since there were few experimental protocols from the same type. This fact did not allow us to conduct a sub-group analysis based on the type of DM intervention.

Given the common limitations among the original studies included, the following issues can be highlighted: (i) there is no report of sample size estimation, (ii) small samples were included, and (iii) risk of bias in non-randomized studies was high. Therefore, future original research should start following specific guidelines for reporting studies as CONSORT. Other points, including specific information about allocation, randomization, blinding, and sample size estimation, must be considered by future studies since existing studies do not report such relevant information.

Regarding possible improvements in methodology, or as future research directions, it would be important to consider personalizing the programs to the skill levels of the players. In addition, it would be important to test the concurrent effect of field-based training sessions (namely, the type of approach by the coach and methodology) to identify possible conflicts of these DM interventions. Finally, further investigation about threshold levels at which DM may be effective or not is necessary. To accomplish this, individual reports on improvements and associations to baseline levels should be considered while aiming to identify eventual responders and non-responders.

Potential practical applications include video-based and questioning-based training, two sessions per week, for youth players. In fact, the combination of more cognitive trainings focused on improving memory-related processes via video feedback and questioning could be beneficial to improve the athletes’ knowledge and better able to differentiate information. In addition, field-based sessions may include skill-based sessions oriented for DM and tactical behavior. A possible combination of these strategies could improve tactical behavior and technical execution, based on the athletes’ needs. In addition, experimental approaches based on imagery-based training could be an interesting approach for contexts in which two sessions are not allowed. Eventually, a few minutes or one session should be tested in future research.

## Conclusion

This systematic review and meta-analysis assessed the effects of DM-based programs on the tactical behavior and technical execution of youth team sports players. DM-based programs (consisting of video-based, imagery-based, or skill-based interventions) yielded significantly greater improvements in tactical behavior compared to control conditions. However, the results should be carefully interpreted due to the heterogeneity of the articles’ overall methodological quality and possible bias influences. The sub-group analysis did not reveal significant changes in the improvements between smaller (<12) and larger (>12) numbers of DM sessions. Despite the reported improvements in tactical behavior, no significant differences were found between the experimental and control groups in terms of technical execution. Intra-group changes revealed significant and beneficial effects of DM-based programs in improving tactical behavior and technical execution, while the control condition did not show significant improvements in either outcome.

## Data Availability Statement

The original contributions presented in the study are included in the article/Supplementary Material, further inquiries can be directed to the corresponding author/s.

## Author Contributions

AS and FC lead the project and wrote and revised the manuscript. JA and HS search the titles and made the methodological assessment and wrote and revised the manuscript. RR-C made the statistical analysis and wrote and revised the manuscript. All authors contributed to the article and approved the submitted version.

## Conflict of Interest

The authors declare that the research was conducted in the absence of any commercial or financial relationships that could be construed as a potential conflict of interest.
